# Pulse wave velocity in South African women and children: comparison between the Mobil-O-Graph and SphygmoCor XCEL devices

**DOI:** 10.1097/HJH.0000000000002976

**Published:** 2021-07-16

**Authors:** Andrea Kolkenbeck-Ruh, Larske Marit Soepnel, Andrew Wooyoung Kim, Sanushka Naidoo, Wayne Smith, Justine Davies, Lisa Jayne Ware

**Affiliations:** a SAMRC/Wits Developmental Pathways for Health Research Unit, Faculty of Health Sciences, University of the Witwatersrand, Johannesburg, South Africa; b Julius Global Health, Julius Center for Health Sciences and Primary Care, University Medical Center Utrecht, Utrecht University, Utrecht, The Netherlands; c Department of Anthropology, Northwestern University, Evanston, Illinois; d Department of Global Health and Population, Harvard T.H. Chan School of Public Health, Boston, Massachusetts, USA; e Hypertension in Africa Research Team (HART); f South African Medical Research Council: Unit for Hypertension and Cardiovascular Disease, North-West University, Potchefstroom, South Africa; g Institute of Applied Health Research, University of Birmingham, Birmingham, UK; h DSI-NRF Centre of Excellence in Human Development, University of the Witwatersrand, Johannesburg, South Africa

**Keywords:** cardiovascular diagnostic technique, child health, pulse wave analysis, Sub-Saharan Africa, vascular stiffness, women's health

## Abstract

**Background:**

Carotid-femoral pulse wave velocity (PWV) is the gold-standard noninvasive measure of arterial stiffness. Data comparing tonometry-based devices such as the SphygmoCor XCEL to simpler brachial-cuff-based estimates of PWV, such as from the Mobil-O-Graph in African populations are sparse. We therefore aimed to compare PWV measured by the Mobil-O-Graph and the SphygmoCor XCEL device in a sample of South African women and children.

**Methods:**

Women (*n* = 85) 29 years [interquartile range (IQR): 29–69] and their children/grandchildren (*n* = 27) 7 years (IQR: 4–11) were recruited for PWV measurement with Mobil-O-Graph and SphygmoCor XCEL on the same day. Wilcoxon signed-rank test, regression analysis, spearman correlation and Bland–Altman plots were used for PWV comparison between devices.

**Results:**

For adults, the SphygmoCor XCEL device had a significantly higher PWV (7.3 m/s, IQR: 6.4–8.5) compared with the Mobil-O-Graph (5.9 m/s, IQR: 5.0–8.1, *P* = 0.001) with a correlation coefficient of 0.809 (*P* ≤ 0.001). Bland--Altman analysis indicated an acceptable level of agreement but significant bias (mean difference PWV: 0.90 ± 1.02 m/s; limits of agreement: −1.10 to 2.90). The odds of having a PWV difference more than 1 m/s decreased with a higher age [odds ratio (OR): 0.95, 95% confidence interval (95% CI) = 0.92–0.98] and increased with greater height (OR: 1.10, 95% CI = 1.01–1.21, *P* = 0.03) in multivariable analysis. In children, the Bland–Altman indicated an excellent level of agreement (−0.03 ± 0.63 m/s; limits of agreement: −1.26 to 1.21), but no correlation was found (*r*_s_ = 0.08, *P* = 0.71).

**Conclusion:**

Particularly in younger and taller women, the Mobil-O-Graph significantly underestimated PWV compared with the SphygmoCor. Although no correlation was found between the two devices for children, further research is required due to the small sample size. Furthermore, the clinical value of both methods in young African populations requires further investigation.

## INTRODUCTION

Cardiovascular disease (CVD) is one of the global leading causes of death and disability [1–3]. Known risk factors for CVD include hypertension, diabetes mellitus and dyslipidaemia [3,4] as well as lifestyle factors such as smoking and obesity [5,6]. Although CVD mortality has declined in developed countries because of better treatment and prevention [7], this is not the case in lower-middle-income countries (LMICs) such as South Africa, where the burden of CVD is greater due to unique healthcare challenges [8]. Improving early identification of at-risk individuals through research of early cardiovascular changes is of paramount importance for delay and prevention of CVD morbidity and mortality [9,10].

Large elastic artery stiffness is an independent predictor of future CVD events in adults [11–13], with increases in arterial stiffness already detectable in children as young as 6 years [14–16]. Carotid–femoral pulse wave velocity (cf-PWV) is considered the gold standard for noninvasive measurement of arterial stiffness, predominantly used in research settings [17,18], and the SphygmoCor Cardiovascular Management System (CvMS) system (AtCor Medical, Sydney, New South Wales, Australia) is well validated and widely used for cf-PWV measurement [13,19]. Of late, a new SphygmoCor device (SphygmoCor XCEL; AtCor Medical), which measures cf-PWV using a partially inflated femoral cuff simultaneously with carotid applanation tonometry, has been developed and validated against the earlier SphygmoCor CvMS device [20], and is therefore also widely used in a research context despite a lack of clinical validation studies for the XCEL device [21,22].

Although the XCEL technique is simpler than the earlier SphygmoCor device (SphygmoCor CvMS; entirely tonometry-based), it still requires relatively skilled operators to acquire high-quality carotid tonometry [23]. More user-friendly and lower-cost ways of estimating pulse wave velocity (PWV) have been proposed based on algorithms using measurements of oscillometric brachial blood pressure (BP) [19,23–25], including the Mobil-O-Graph (I.E.M. GmbH, Aachen, Germany). The Mobil-O-Graph is an automated oscillometric brachial ambulatory BP monitoring device which estimates PWV with an inbuilt ARCSolver (Austrian Institute of Technology, Vienna, Austria) algorithm [25]. The algorithm incorporates age, central SBP and parameters from pulse wave analysis to produce an estimate of aortic PWV [19,25–27].

Mobil-O-Graph-estimated PWV measures have been validated against direct intra-arterial measurement in Austrian populations [25,27]. Studies comparing the Mobil-O-Graph with the SphygmoCor CvMS device in high-income countries (HIC), including Denmark [28] and Spain [29], show the devices are comparable. However, similar research from a LMIC population in Uruguay [30] suggests that 24-h ambulatory Mobil-O-Graph measures may underestimate PWV in adults. There is scarce evidence for how the two devices compare in African adults and children despite the surge in CVD in this population. Thus, the aim of our study was to compare PWV measured by both the Mobil-O-Graph and the SphygmoCor XCEL device in a LMIC population in South Africa in both women and children.

## MATERIALS AND METHODS

### Study population

The current study was part of a cross-sectional assessment of vascular health in an existing birth cohort known as the Birth-to-Twenty (BT20) study started in 1990 (described previously [31]), which now spans across three generations. Participants were invited between August 2019 and March 2020 to attend a data collection appointment for cardiovascular measurements as part of the Intergenerational Vascular Health Study in Soweto, at which arterial stiffness was assessed using two devices, the Mobil-O-Graph and SphygmoCor XCEL. Participants were recruited for the study based on the following inclusion criteria: previously enrolled in the BT20 study, two successful measurements of arterial stiffness for each device, the Mobil-O-Graph and SphygmoCor XCEL. Participants that were pregnant or had a mid-upper arm circumference greater than 38 cm were excluded from this analysis. Trained researchers who spoke the participant's home language explained the study and all participants provided written informed consent prior to taking part. For children, the mother of the child provided written consent that her child may take part in the study, with children older than 7 years additionally providing written assent to take part. The study was conducted according to the principles of the Helsinki declaration [32] and the Human Research Ethics Committee (Medical) of the University of the Witwatersrand approved the protocol (M190263).

### Clinical, demographic, anthropometric and blood pressure measurements

A health questionnaire was administered to obtain demographic information including current self-reported tobacco use (smoking and smokeless tobacco), self-reported alcohol consumption and medical history, including self-reported diabetes mellitus or high cholesterol and current medical treatment for these conditions. Thereafter, anthropometry including height, weight, waist circumference and mid-upper arm circumference (MUAC) was measured in triplicate on the same day as Mobil-O-Graph and SphygmoCor XCEL measurements. Height was measured to the nearest 0.1 cm using a stadiometer (Holtain, Crymrych, UK). Weight was assessed using an electronic scale, to the nearest 0.1 kg (SECA, Hamburg, Germany). Waist circumference was measured to the nearest 0.1 cm at the mid-point between the lower costal margin and the level of the anterior superior iliac crests and MUAC was measured to the nearest 0.1 cm at the midpoint between the acromion and the olecranon process. The average MUAC was used to determine the correct brachial cuff size for blood pressure (BP) measurements. Following the anthropometric measurements, brachial BP was measured using Omron MIT5 (for adults) and Omron HBP-1300 (for children) (Omron Healthcare, Kyoto, Japan). This was used as an independent BP reading to be used in conjunction with the health questionnaire for hypertension status. Following the International Society of Hypertension (ISH) guidelines [33], the participants were seated and asked to rest for at least 5 min. The researchers measured the participant's BP on their right arm three times, consistent with the cuff position for Mobil-O-Graph and SphygmoCor XCEL, with a 2-min rest interval between the measurements. If a difference between the second and third measure was greater than 5 mmHg, a fourth measure was performed, so that the average of the two reading within a 5 mmHg range was used for analysis.

### Arterial stiffness measurements

Both Mobil-O-Graph and SphygmoCor Xcel measurements were performed on the same day and in a quiet room within an average of 60 (60.4 ± 29.6) min of each other. In total, four trained operators performed the PWV measurements. The inter-observer variability between operators was not more than 0.5 m/s. All participants had not eaten, drank or smoked for at least 3 h prior to measurements. Following anthropometric measurements, the participants laid supine for at least 10-min prior to measurements. Device order varied throughout the day and study. For SphygmoCor measurements, we used the SphygmoCor XCEL device (AtCor Medical, Naperville, Illinois, USA) to measure peripheral BP and PWV. Peripheral BP was obtained by partially inflating a size appropriate cuff over the right brachial artery approximately midway between the shoulder and the elbow. This was needed as an input variable along with height for PWV measurement. Thereafter, to assess carotid PWV, following input of the participant's peripheral BP, height, sex and date of birth, carotid pulse waves were measured by applanation tonometry (high-fidelity micromanometer; Millar Instruments) and a partially inflated cuff simultaneously obtained femoral pulse waves over the femoral artery at the leg midway between the hip and the knee. These simultaneously captured carotid and femoral waveforms, over a preset time of 10 s. The distance between the carotid and femoral arteries was measured with a tape measure using the direct distance method. This chosen method, as recommended by an expert consensus on the measurement of aortic stiffness in 2012 [17], uses 80% of the distance from the carotid pulse to the femoral pulse. The SphygmoCor software determines the PWV using this corrected distance and the pulse wave transit time (the time delay between the carotid and femoral pulse waves). The SphygmoCor device's inbuilt quality control criteria were applied. Each PWV measure was conducted at least twice, with 1-min rest between each measurement, and if the difference between PWV results was greater than 0.5 m/s, a third PWV measurement was performed. Overall, following a standard protocol (setup, 10-min rest, PWA followed by PWV), it took approximately 30 min to obtain two valid PWV measurements. Thereafter the two PWV values that fell within the 0.5 m/s range was used to calculate the average PWV measure.

For Mobil-O-Graph measurements, participants were fitted with the Mobil-O-Graph (I.E.M. GmbH, Germany) and weight, height, smoking status, sex and date of birth were entered into the Mobil-O-Graph software (I.E.M., HMS Client-Server version 5.2). After the participants had rested for 10 min in the supine position, an appropriate size brachial cuff for the right upper arm circumference was placed 2 cm above the elbow on the participant's right side. This device performs a brachial oscillometric BP and immediately afterwards it records the pulse waves at the level of the brachial artery. For calibration of the brachial pulse waveforms, the system uses the oscillometric brachial SBP and brachial DBP. Then, the aortic pulse waveform is generated with the ARCSolver algorithm generalized transfer function and provides an indirect estimate of the aortic PWV through mathematical models taking into account age and several parameters obtained by pulse wave and wave-separation analysis [27,34–36]. Following the same criteria used with the SphygmoCor, measurement was repeated after 1-min rest, and if the difference between the PWV results was greater than 0.5 m/s, a third measurement was performed. In general, obtaining two PWV measurements using Mobil-O-Graph took approximately 20 min. Thereafter, the two PWV values that fell within the 0.5 m/s range was used to calculate the average PWV measure.

### Data management

In adults, BMI was calculated as weight in kilograms divided by the square of height in meters and categorized according to WHO classification as follows: less than 18.5 kg/m^2^ as underweight; 18.5–24.9 kg/m^2^ as normal; 25–29.9 kg/m^2^ as overweight; and at least 30 kg/m^2^ as obese [37]. In children, age and sex-adjusted *z*-scores for BMI were calculated using the WHO reference and children were categorized as overweight if their BMI *z*-score was between 1 and 2, and obese if their BMI *z*-score was more than 2 [37]. Waist to height ratio (WHtR) was calculated as the waist circumference (cm) divided by height (cm), and a WHtR of ≥0.5 was classified as ‘high’ [38]. For BP measurements, the first measurement was discarded and we averaged the second and third measurement. In adults, hypertension was defined as a BP at least 140 mmHg systolic or at least 90 mmHg diastolic or currently taking antihypertensive medication [33]. For children, hypertension was defined as being above the 95th percentile using standards adjusted for age and height following the American Academy of Pediatrics Clinical Practice 2017 guidelines [39]. For each device, as recommended by the ARTERY society guidelines [40], only PWV readings within 0.5 m/s of each other were used and the average calculate for statistical analysis.

### Statistical analysis

SPSS statistics 25.0 (IBM, Chicago, Illinois, USA) was used for statistical analysis. Distribution of the samples was tested using visual inspection of histograms and the Shapiro–Wilks test. As the data were not normally distributed, data were reported as median and interquartile range (IQR) for continuous data and as absolute numbers and percentages for categorical data.

The Wilcoxon signed-rank test was used to compare the PWV measurements from the two devices. A Spearman's correlation coefficient was used to evaluate the correlation between the Mobil-O-Graph's PWV and SphygmoCor Xcel's PWV results. Univariate regression analysis was performed to evaluate the relationship between PWV and age, height, weight, BMI, WHtR, diabetes, hypertension, alcohol and tobacco use, MUAC, SBP, DBP and mean arterial pressure (MAP) for each device within adults. In children, univariate regression analysis was performed to evaluate the relationship between PWV and age, sex, height, weight, BMI, WHtR, MUAC, hypertension, SBP, DBP and MAP for each device. We then performed multiple regression analysis to determine independent predictors of PWV, testing the normality of residuals. Variables considered for entry into the model were chosen based on significant univariate relationships with PWV. We further excluded variables showing multicollinearity. In the case where a statistically significant difference was found between PWV results from the two devices, logistic regression was performed to examine the relationship between the absolute PWV difference greater than one meter per second (>1 m/s) and the following variables: age, WHtR, WHtR at least 0.5, weight, height, BMI, MUAC, hypertension, diabetes mellitus, SBP, DBP, MAP, current tobacco and alcohol use. This was followed by multivariable logistic regression for those variables with a *P* value less than 0.05 in univariate analysis after checking for multicollinearity using a variance inflation factor of less than five [41]. When an statistically significant association was found in univariate logistic regression analysis (Table S1) between a variable (e.g. height) and a PWV difference of more than 1 m/s between devices, receiver operator characteristic (ROC) curves were generated to determine the threshold with the highest sensitivity and specificity to predict a PWV difference of more than 1 m/s. Examining the area under the curve (AUC) and producing the Youden index using the following formula: sensitivity + specificity − 1, we produced threshold values with the maximum obtained value corresponding to the optimal cut-off point [39]. Thereafter, logistic regression was used to determine if the calculated thresholds significantly predicted a PWV difference of more than 1 m/s between the two devices.

Together with the Bland--Altman plot, a one-sample *t*-test using the mean difference between the two devices within adults and children respectively was calculated to determine if the mean PWV between the two devices differed significantly from zero. Agreement between the two devices for PWV measurement was analysed using the Bland and Altman method [42], and interpreted according to Artery Society Guidelines cut-offs for agreement [43]. Statistical significance for all analyses was set at *P* value less than 0.05. With 85 adults and 27 children, the sample had 84% power to detect a difference of 0.25 m/s (SD 1.3; α = 0.05) between the devices.

## RESULTS

### Characteristics of the participants

**FIGURE 1:**
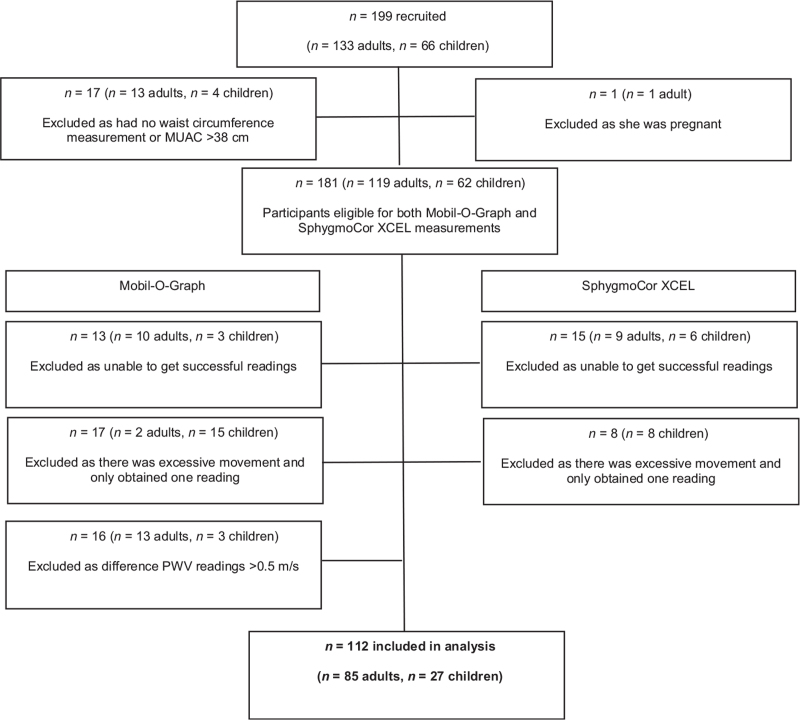
Study flow diagram.

Of the 199 participants recruited, we included 112 (56.3%) in the analysis. Of the 43.7% excluded, 9% (*n* = 18) were excluded as they were pregnant, had no waist circumference measurement or a MUAC more than 38 cm (Fig. 1). Of those eligible for both Mobil-O-graph and SphygmoCor measurement (*n* = 181), 69 (38.1%) were excluded for unsuccessful Mobil-O-Graph measurement (*n* = 46, 25.4%), and/or SphygmoCor XCEL measurement (*n* = 23, 12.7%). Examination of the cohort with no successful measurements showed that these adults were significantly more obese, had a higher MUAC, waist circumference and WHtR (*P* < 0.01), while the children had a significantly smaller MUAC and a lower BMI (*P* < 0.05) as compared to the cohort with successful measurements. Of the participants with successful measurements (*n* = 112), 13.4% required a third or fourth measurement to achieve two measures within the acceptable limits of variation (<0.5 m/s).

In the analytical sample, the median age for adult women was 29 years (IQR: 29–55 years), while for children it was 7 years (IQR: 7–9 years; 52% female) (Table 1). The median BMI was 30.2 kg/m^2^ (IQR: 25.9–34.5 kg/m^2^) and 16 kg/m^2^ (IQR: 14.7–18.0 kg/m^2^) for adults and children, respectively, with 51.8% (*n* = 44) of adults and 7.4% (*n* = 2) of children being obese. Of the 85 adults, 8% were tobacco users (smoking and smokeless tobacco) and 52% consumed alcohol. A small percentage, 12 and 1% of adults self-reported having high cholesterol and type II diabetes mellitus respectively. Although all self-reported diabetic individuals were on treatment, only 30% of adults with high cholesterol were on treatment. Approximately, one-third (29.4%) of adults were hypertensive with 82% reported to be on treatment. Nearly half of children were hypertensive (48%).

**TABLE 1:** Study sample characteristics

	Total group (*n* = 112)	Adults (*n* = 85)	Children (*n* = 27)
Age (years)	29.0 (29.0–51.8)	29.0 (29.0–55.0)	7.0 (7.0–9.0)
Women, *n* (%)	99 (88.4)	85 (100.0)	14 (51.8)
Anthropometry			
Height (cm)	155.1 (147.0–161.9)	159.0 (154.1–162.5)	124.0 (120.0–137.0)
Weight (kg)	69.7 (45.9–81.2)	76.0 (66.1–88.9)	26.7 (21.1–30.1)
BMI (kg/m^2^)	27.6 (20.2–33.6)	30.2 (25.9–34.5)	16.0 (14.7–18.0)
Underweight (BMI ≤ 18 or <−2SD in children), *n* (%)	3 (2.7)	2 (2.4)	0 (0)
Normal (BMI: 18–25 or −2 to 1SD in children), *n* (%)	33 (29.5)	15 (17.6)	21 (77.8)
Overweight (BMI: 25–30 or 1–2SD in children), *n* (%)	27 (24.1)	24 (28.2)	4 (14.8)
Obese (BMI ≥ 30 or >2SD in children), *n* (%)	49 (43.7)	44 (51.8)	2 (7.4)
Mid-upper arm circumference (cm)	26.4 (20.0–32.0)	29.5 (24.1–32.3)	16.3 (14.6–19.2)
Waist circumference (cm)	75.2 (57.3–90.0)	81.0 (69.0–93.2)	50.9 (43.7–56.3)
Waist: Height ratio (WHtR ≥ 0.5), *n* (%)	49 (43.7)	46 (54.1)	3 (11.1)
Sitting blood pressure (Omron)			
SBP (mmHg)	111 (105–122)	112 (105–125)	110 (105–117)
DBP (mmHg)	77 (72–83)	79 (73–85)	73 (68–78)
Mean arterial pressure (mmHg)	88 (83–94)	90 (84–98)	86 (78–91)
Heart rate (bpm)	77 (66–87)	73 (63–83)	87 (85–100)
Hypertensive, *n* (%)	38 (33.9)	25 (29.4)	13 (48.1)
Medical history			
Previously diagnosed diabetes mellitus, *n* (%)	–	1 (1.2)	–
Previously diagnosed high cholesterol, *n* (%)	–	10 (11.8)	–
Tobacco and alcohol use			
Current tobacco use, *n* (%)	–	7 (8.2)	–
Current alcohol use, *n* (%)	–	44 (51.8)	–

Data are presented as median and interquartile range, unless otherwise indicated. BP, blood pressure; bpm, beats per minute; WHtR, waist to height ratio.

### Comparison of pulse wave velocity between the Mobil-O-Graph and SphygmoCor XCEL devices in adults

**FIGURE 2:**
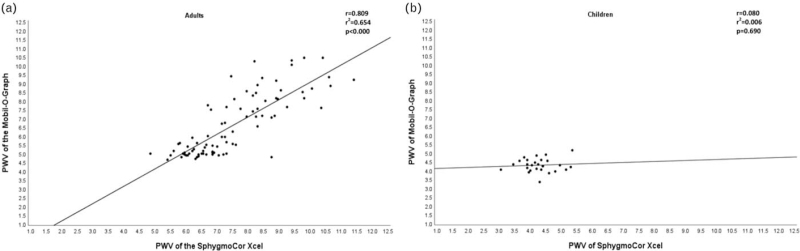
Scatter plot of the association between pulse wave velocity of the SphygmoCor XCEL device and the PWV of the Mobil-O-Graph device in adults (a) and in children (b).

**FIGURE 3:**
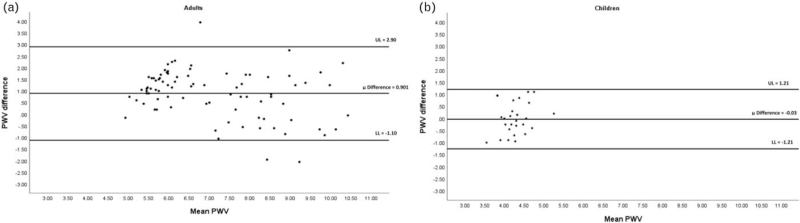
Bland--Altman plot for the limit of agreement of the pulse wave velocity (PWV) between the SphygmoCor XCEL and Mobil-O-Graph devices in adults (a) and in children (b). μ Difference, mean difference; LL, lower limit; UL, upper limit.

Within adults, PWV measured using the Mobil-O-Graph was significantly lower (median: 5.9 m/s, IQR: 5.0–8.1 m/s) than the PWV measured using the SphygmoCor XCEL (median: 7.3 m/s, IQR: 6.4–8.5 m/s; *P* < 0.0001; Table 2). The Spearman correlation showed a strong, significant correlation between the PWV measured with the Mobil-O-Graph and the PWV measured with the SphygmoCor XCEL (*r*_s_ = 0.809, *P* < 0.0001) (Fig. 2a). The results from the Bland--Altman show acceptable agreement as most of the points fall within the upper and lower agreement limits [44] (Fig. 3a). However, there is evidence of bias as the mean difference between the two devices differed significantly from zero (mean difference = 0.9001, 95% CI: 0.680–1.121, *P* = 0.0001). On the basis of the Bland--Altman plot, the bias seems to be in the higher values.

**TABLE 2:** Peripheral blood pressure and pulse wave velocity measurements from the Mobil-O-Graph and SphygmoCor XCEL devices for the total sample, within adults and within children

Total sample *(n* *=* 112)	Mobil-O-Graph	SphygmoCor	*P*
Brachial SBP	115 (108–129)	115 (106–129)	0.093
Brachial DBP	74 (68–87)	72 (65–81)	0.001
Heart rate	69 (61–77)	69 (62–77)	0.697
Pulse wave velocity	5.2 (4.8–7.6)	6.7 (5.4–8.2)	**<0.0001**
Adults (*n* = 85)			
Brachial SBP	120 (111–134)	121 (111–134)	0.494
Brachial DBP	78 (71–93)	74 (68–85)	**0.001**
Heart rate	67 (59–74)	66 (60–73)	0.903
Pulse wave velocity	5.9 (5.0–8.1)	7.3 (6.4–8.5)	**<0.0001**
Children (*n* = 27)			
Brachial SBP	108 (98–113)	104 (97–108)	**0.012**
Brachial DBP	65 (59–69)	63 (58–67)	0.904
Heart rate	81 (73–90)	79 (73–87)	0.449
Pulse wave velocity	4.3 (4.1–4.6)	4.3 (3.9–4.6)	0.716

Data are represented as median and interquartile range. *P* values are for comparison between SphygmoCor XCEL and Mobil-O-Graph measurements. *P* < 0.05 was significant, marked in bold.

Univariate regression analysis (Table 3) showed age, BMI, waist circumference, WHtR and WHtR at least 0.5, arm circumference, SBP, DBP, MAP and presence of hypertension as well as alcohol consumption to be associated with Mobil-O-Graph PWV. Whereas age, WHtR, presence of diabetes, SBP, DBP, MAP and presence of hypertension as well as alcohol consumption to be associated with PWV by SphygmoCor XCEL. In multivariate regression analysis, in which we excluded variables showing multicollinearity, only age remained significantly associated with PWV for Mobil-O-Graph, while age and MAP remained significantly associated with PWV for SphygmoCor (Table 3).

**TABLE 3:** Regression analysis to evaluate the relationship between pulse wave velocity and clinical factors from each device in adults and children

Adults *(n* *=* 85)	PWV using Mobil-O-Graph				PWV using SphygmoCor XCEL			
Characteristics	Univariate		Multivariable		Univariate		Multivariable	
	ß (SE)	*P*	ß (SE)	*P*	ß (SE)	*P*	ß (SE)	*P*
Age	0.925 (0.005)	**<0.0001** ^a^	0.753 (0.030)	**0.001**	0.773 (0.007)	**<0.0001** ^a^	0.553 (0.008)	**<0.0001**
Body height	−0.184 (0.031)	0.091			−0.065 (0.025)	0.557		
Body weight	0.210 (0.012)	0.054			0.141 (0.010)	0.198		
BMI	0.268 (0.268)	**0.013** ^a^	0.239 (0.062)	0.391	0.154 (0.024)	0.159		
Waist circumference	0.272 (0.011)	**0.012**			0.181 (0.010)	0.097		
WHtR	0.304 (1.768)	**0.005** ^a^	0.125 (5.304)	0.786	0.214 (1.487)	**0.049** ^a^	−0.072 (0.947)	0.296
WHtR ≥ 0.5	0.246 (0.368)	**0.024**			0.112 (0.310)	0.307		
Arm circumference	0.226 (0.032)	**0.037** ^a^	−0.419 (0.076)	0.284	0.133 (0.027)	0.226		
Diabetes mellitus	0.172 (1.730)	0.115			0.237 (1.399)	**0.029** ^a^	0.073 (0.857)	0.266
Hypertension	0.665 (0.310)	**<0.0001** ^a^	−0.128 (0.654)	0.553	0.647 (0.260)	**<0.0001** ^a^	0.132 (0.289)	0.159
Smoking	−0.108 (0.685)	0.324			−0.055 (0.564)	0.616		
Alcohol use	−0.338 (0.357)	**0.002** ^a^	−0.156 (0.483)	0.361	−0.352 (0.291)	**0.001** ^a^	−0.040 (0.193)	0.559
Brachial SBP	0.685 (0.007)	**<0.0001**			0.639 (0.006)	**<0.0001**		
Brachial DBP	0.611 (0.013)	**<0.0001**			0.620 (0.010)	**<0.0001**		
MAP	0.670 (0.010)	**<0.0001** ^a^	0.208 (0.013)	0.228	0.654 (0.009)	**<0.0001** ^a^	0.254 (0.010)	**0.008**
Heart rate	−0.106 (0.016)	0.334			0.051 (0.014)	0.645		
Children (*n* = 27)								
Age	0.327 (0.040)	0.096			0.076 (0.060)	0.706	–	–
Sex	−0.071 (0.147)	0.726			0.028 (0.210)	0.896	–	–
Body height	0.413 (0.007)	**0.032**	−0.507 (0.014)	0.183	0.141 (0.011)	0.483	–	–
Body weight	0.535 (0.008)	**0.004**	0.805 (0.018)	**0.047**	0.046 (0.013)	0.820	–	–
BMI	0.526 (0.055)	0.066			−0.110 (0.042)	0.583	–	–
Waist circumference	0.420 (0.006)	**0.029**	0.819 (0.020)	0.165	−0.041 (0.010)	0.840	–	–
WHtR	0.284 (1.198)	0.152			−0.128 (1.767)	0.525	–	–
WHtR ≥ 0.5	0.172 (0.231)	0.390			−0.074 (0.334)	0.716		
Arm circumference	0.406 (0.016)	**0.036**	−0.647 (0.050)	0.276	−0.043 (0.025)	0.830	–	–
Brachial SBP	0.456 (0.006)	**0.017**	0.449 (0.006)	**0.015**	−0.058 (0.010)	0.773	–	–
Brachial DBP	0.211 (0.010)	0.291			−0.150 (0.014)	0.455	–	–
MAP	0.360 (0.010)	0.065			−0.128 (0.015)	0.525	–	–
Heart Rate	−0.090 (0.006)	0.655			0.088 (0.008)	0.662	–	–

β, beta coefficient; MAP, mean arterial pressure; PWV, pulse wave velocity; SE, standard error; WHtR, waist to height ratio.

a Variables used in multivariable regression analysis to avoid multicollinearity bias. *P* < 0.05 was significant, marked in bold.

For the outcome of having an absolute difference in PWV more than 1 m/s between devices, univariate logistic regression showed age (OR 0.947, *P* = 0.002), body height (OR 1.107, *P* = 0.018) and WHtR ≥ 0.5 (OR 0.146, *P* = 0.027) were significantly associated with having an absolute mean PWV difference more than 1 m/s (supplementary table S1). The results of the multivariate logistic regression, including those variables that were statistically significant in univariate analysis, are summarized in Table 4. Due to the association between body height and WHtR at least 0.5, the variables were analysed in two separate models. When including age and WHtR at least 0.5 within the model, only age (OR 0.952, *P* = 0.006) was significantly associated with having an absolute mean PWV difference more than 1 m/s. In contrast, when including body height and age, both were significantly associated with having and an absolute mean PWV difference more than 1 m/s (Table 4).

**TABLE 4:** Multiple logistic regression analysis to evaluate the role of clinical factors in affecting the difference in pulse wave velocity between devices of > 1 m/s, respectively, in adults

Independent variable	Odds ratio	95% CI	*P*	*R* ^2^
Model I				0.173
Age	0.948	0.916–0.982	**0.003**	
Body height	1.103	1.009–1.205	**0.031**	
Model II				0.143
Age	0.952	0.920–0.986	**0.006**	
Waist to height ratio ≥ 0.5	0.138	0.184–1.264	0.138	

CI, confidence interval. *P* < 0.05 was significant, marked in bold, *R*^2^ by Cox and Snell.

**FIGURE 4:**
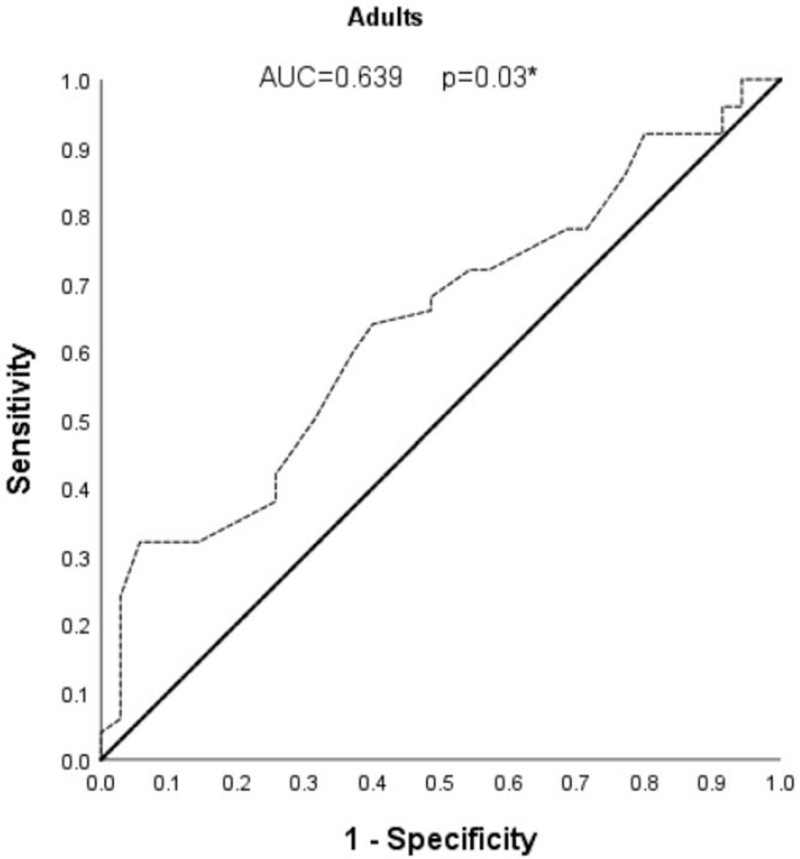
Receiver operator characteristic curve showing the accuracy of height [area under the curve (AUC; *P* = 0.03) shown as a dotted line] to predict a PWV difference of >1 m/s between devices in (*n* = 85) adult women.

As shown in Fig. 4, to estimate the threshold at which height predicts a difference of PWV more than 1 m/s between devices, we performed ROC curve analysis. The area under the ROC curve (AUC) was significantly associated with a PWV more than 1 m/s between the two devices (*P* = 0.03). A height cut-off of 157.5 cm (64% sensitivity, 40% specificity) was determined as the optimal cut-off point using the Youden index. Height values above this cuff-off level was significantly associated with a PWV more than 1 m/s difference between the two devices (OR: 2.667, 95% CI 1.096–6.489, *P* = 0.031).

### Comparison of pulse wave velocity between the Mobil-O-Graph and SphygmoCor XCEL devices in children

In contrast, in children, there was no difference in median PWV measured by the Mobil-O-Graph (4.3 m/s, IQR 4.1–4.6 m/s) and the PWV measured by the SphygmoCor XCEL (4.3 m/s, IQR 3.9–4.6 m/s; *P* = 0.716; Table 2). The mean difference between the two devices did not differ significantly from zero (mean difference = −0.028, 95% CI: −0.277 to 0.221, *P* = 0.820), thus no bias was observed. Furthermore, no correlation was found between the PWV measured with the Mobil-O-Graph and the PWV measured with the SphygmoCor XCEL (*r*_s_ = 0.080, *P* = 0.712) (Fig. 2b), and the Bland--Altman analysis showed that there was an excellent level of agreement (mean difference −0.028 m/s, lower limit – upper limit: −1.260 to 1.205) between the two device measurements of PWV (Fig. 3b). Body weight and SBP were independently associated with PWV from the Mobil-O-Graph after correcting for height, waist circumference and MUAC (Table 3). For the SphygmoCor XCEL, there were no associations between any of the variables or BP measures and PWV (all *P* > 0.05).

## DISCUSSION

In the present study, we compared the estimated aortic PWV from Mobil-O-Graph to cf-PWV measurement from SphygmoCor XCEL in African women and children. Our findings show an acceptable level of agreement between the two devices according to the ARTERY Society guidelines [43]. However, significant bias was detected towards lower PWV readings by the Mobil-O-Graph. In contrast, among prepubescent children (4–11 years), there was no statistical difference in the mean PWV values and an excellent level of agreement [43] between the devices. However, although one would expect a high correlation between the two devices [44,45], this was not observed in the current study. Considering that there was a poor correlation between PWV measurement and that our sample size was small (*n* = 27), conclusions regarding the validity of PWV by the Mobil-O-Graph in children need to be confirmed in future studies with larger sample sizes.

The Mobil-O-Graph, with the simple-to-apply brachial cuff, is an attractive alternative to devices requiring unique skill to apply applanation tonometry, accurately, over arterial pulses. The ARCSolver algorithm (used by the Mobil-O-Graph) uses brachial pulse wave characteristics, age and SBP to estimate PWV [27,34–36], and validation against invasive PWV measures in HIC has been promising [25,27]. However, most of these studies have been conducted in older age groups [18,25,27,36], and Mobil-O-Graph has not, to our knowledge, been compared with another widely used PWV device in an urban African setting.

Results from the ROC curve and logistic regression showed that adult women with a height greater than 157.5 cm have twice the odds of a PWV difference more than 1 m/s. With an estimated national average of 157.8 cm [46], this potentially impacts a large proportion of South African women. However, the sensitivity and specificity of the ROC curve were relatively low, and more future research is needed to evaluate the precise impact within an African population. Despite this, our results suggest that height has a significant impact, with taller participants in our study having greater odds of a PWV difference more than 1 m/s between devices. In South Africa, early childhood stunting is common, with a prevalence at age 2 between 18 and 34% [47,48]. Stunting in early childhood seems to impact adult stature largely through decreased relative leg-length, as legs grow fastest between birth and puberty [49–52]. Therefore, trunk vs. leg length proportions may differ between our population and reference populations from HIC. With the SphygmoCor XCEL device, measuring individual path distance may account for this, but no such assessment of path length or trunk size is used by Mobil-O-Graph, and only total height is entered [18]. This may explain similar findings from another LMIC population in Uruguay, which found slightly lower PWV by laboratory based and 24-h ambulatory Mobil-O-Graph compared to the original SphygmoCor in adults aged 45–50 years [30]. Although more recent data have shown a reduced level of stunting (<5 years) in Uruguay, in the 1980s, stunting levels were similar to South Africa [53,54]. Interestingly, a recent study by Salvi *et al.*[55] in a population with Marfan Syndrome, which is characterized by greater relative leg-length, also found that Mobil-O-Graph had poor agreement with tonometry-based cf-PWV. Therefore, we speculate that population-specific differences in the pulse-wave path distance, potentially as a result of nutritional insults or disease, may influence the accuracy of the Mobil-O-Graph algorithm.

A study comparing, amongst others, the original SphygmoCor CvMS and Mobil-O-Graph to invasive PWV, showed that Mobil-O-Graph results could largely be predicted by participant age and SBP [19]. As a result, the Mobil-O-Graph algorithm for PWV *may not account for* factors that play a role in arterial stiffness but are not directly related to SBP or age. One possible example of this is early vascular ageing, as suggested by results from a PWV-method comparison study in patients with Marfan syndrome [55]. In our population, a recent South African study indicated that arteriosclerotic changes were prevalent as early as 20 years of age, which may not be predicted by traditional risk factors such as age or BP [56]. This early vascular ageing may prevent accurate identification of individuals with a higher PWV, where age and SBP-based algorithms are used, requiring further research into the clinical significance of these findings.

The accuracy of our results from the SphygmoCor XCEL PWV measurement should also be considered. The original SphygmoCor device has been widely used in research of South African populations [57–59], after being validated against invasive procedures [25,60]. The newer SphygmoCor XCEL has been validated and compared against the original SphygmoCor [23] including youth (6–20 years) [22], but it has not, to our knowledge, been compared with invasive measures, and no clinical outcome studies using the XCEL device are yet available [18]. The method to measure path distance from the carotid to the femoral pulse for cf-PWV is debated, and, as a result, methods may vary amongst research groups. Although a key validation paper for SphygmoCor XCEL uses the ‘subtraction method’, an expert consensus published in 2012 recommended that 80% of the direct measurement from the carotid artery to the femoral artery (the direct method X 0.8) is the most accurate measure of the actual distance when compared with MRI [17,18]. However, this direct method for cf-PWV was found to overestimate aortic PWV measured invasively, particularly in younger adults [25]. Therefore, overestimated results from the SphygmoCor XCEL device, used in younger adult patients using the direct x 0.8 path distance, may have contributed to the discrepancy we found with the Mobil-O-Graph.

There are multiple strengths to this study. Firstly, the ability to perform measures using Mobil-O-Graph and SphygmoCor XCEL under similar conditions on the same day under strict protocols limits the potential confounding of results. The measurements were conducted so as to not create an order effect and the average time from the first device to the second was an hour.

A limitation of the present study is that we could not validate the PWV findings from the two devices against invasive PWV measurement. As recommended by the ARTERY society [40] and the American Heart Association [26], the true gold standard for aortic stiffness is invasive PWV from the ascending aorta to the bifurcation into the common iliac arteries. Although invasive studies are technical, expensive and potentially unethical in healthy populations, further investigations are needed in which these devices are validated and tested against invasive PWV measurement within African and LMIC populations. A second limitation is that we were not always able to perform both measures before midday, which may have caused circadian rhythms to impact results. However, as the order of the device used changed between participants, this should not have introduced bias for a particular device. Another limitation was the lack of continuous ages within the adult group. As this study was a sub study of an intergenerational study, in which the second-generation adults are all 29 years old, the ages in adults are clustered around 29 and 45–65 years. Although age was incorporated in the multiple logistic regression models, this clustering prevented us from determining an age at which the devices become acceptably comparable, and the difference in age may reflect another clinical characteristic in the 29-year-old participants that we were not able to correct for explicitly. Lastly, there were no adult males included, significantly limiting the generalizability of our results and our ability to draw conclusion on whether the devices may be comparable in men.

In conclusion, although the devices were comparable in both children and adult women, the observed significant bias within young adult women, with SphygmoCor XCEL resulting in higher values compared to the Mobil-O-Graph, is concerning. The earlier onset of cardiovascular morbidity and risk in South African adults [56] highlights the importance of accurate noninvasive and accessible measures for arterial stiffness in this group. Our results suggest, however, that validity of both devices (Mobil-O-Graph algorithm and SphygmoCor XCEL) may require further investigation in African populations, particularly to determine if stunting and earlier vascular ageing impact the accuracy of results. In addition, recent research by Hametner *et al.*[61] has shown that both invasive and noninvasive measurements of PWV are not only suitable measures of arterial stiffness but also are predictors of cardiovascular events and mortality. Therefore, further research is needed to confirm the accuracy and potential predictability of these PWV measures by the Mobil-O-Graph and SphygmoCor XCEL in the South African population, including children, women and men.

## ACKNOWLEDGEMENTS

This study would not have been possible without the voluntary collaboration of the participants and the excellent technical assistance of Betty Nembulu, Mam Meikie Priscilla Matabane, Monica Muti and Irene Masuluke.

This research was funded in whole, or in part, by the Wellcome Trust [Grant number: 214082/Z/18/Z]. For the purpose of Open Access, the author has applied a CC BY public copyright licence to any Author Accepted Manuscript version arising from this submission. This research is also supported the Faculty Research Committee (FRC), University of the Witwatersrand.

### Conflicts of interest

None disclaimers.

## Supplementary Material

Supplemental Digital Content
