# Supplementary material for: Pulse wave velocity in South African women and children: comparison between the Mobil-O-Graph and SphygmoCor XCEL devices
Source: J Hypertens. 2021 Jul 16;40(1):65–75. doi: 10.1097/HJH.0000000000002976 (PMC8654263; doi:10.1097/HJH.0000000000002976)
Supplement: Supplemental Digital Content [file jhype-40-065-s001.docx]

**Table S1.** Univariate logistic regression analysis to evaluate the role of clinical characteristics and haemodynamic factors in affecting the absolute PWV difference greater than 1 m/s in adults (n=85).

| ***Characteristics*** | ***OR (95% CI)*** | ***p*** |
| --- | --- | --- |
| Age | 0.947 (0.915-0.979) | **0.002*** |
| Body height | 1.107 (1.017-1.204) | **0.018*** |
| Body weight | 0.995 (0.6991.022) | 0.722 |
| Body mass index | 0.952 (0.888-1.021) | 0.168 |
| Waist to height ratio | 0.029 (0.000-2.313) | 0.113 |
| WHtR≥0.5 | 0.360 (0.146-0.891) | **0.027*** |
| Arm circumference | 0.941 (0.870-1.017) | 0.126 |
| Diabetes mellitus | 0.363 (0.032-4.151) | 0.415 |
| Hypertension | 0.423 (0.163-1.095) | 0.076 |
| Smoking | 4.636 (0.533-40.355) | 0.165 |
| Alcohol consumption | 1.243 (0.523-2.953) | 0.622 |
| Brachial systolic blood pressure | 0.983 (0.96.-1.007) | 0.161 |
| Brachial diastolic blood pressure | 0.982 (0.947-1.018) | 0.982 |
| Heart Rate | 0.980 (0.949-1.012) | 0.224 |
| Mean arterial pressure | 1.020 (0.982-1.060) | 0.299 |
| PWV, pulse wave velocity; WHtR, waist to height ratio; CI, confidence levels, OR, odds ratio, SE, standard error. p<0.05 was significant. | | |
